# Supplementary material for: Dynalign II: common secondary structure prediction for RNA homologs with domain insertions
Source: Nucleic Acids Res. 2014 Nov 21;42(22):13939–48. doi: 10.1093/nar/gku1172 (PMC4267632; doi:10.1093/nar/gku1172)
Supplement: SUPPLEMENTARY DATA [file supp_gku1172_nar-02021-z-2014-File012.zip › manual/Overview/Installation_Instructions_Linux.html]

RNAstructure Installation and Overview: Installation Instructions for Linux Systems


|  |  |  |
| --- | --- | --- |
|  | RNAstructure Installation and Overview Installation Instructions for Linux Systems | - Contents - Index |
| Installation of the RNAstructure text interfaces and GUI proceed in a similar fashion on both 32 and 64-bit Linux systems. This page gives details on how to install the prebuilt package. For information on how to build the package, please refer to the Building Instructions and Building Requirements pages. To install the JAVA graphical user interface (GUI), follow these steps:   1. Make sure JAVA 1.6 or higher is installed on your system. It is crucial that the SUN software be used, and it can be found here.- Download the JAVA GUI for either 32 or 64-bit from the Mathews Lab RNAstructure page.    - The GUI is supplied as a GZipped tarball, so the files must be extracted. Move the tarball to the desired directory and execute the command *tar -xzvf RNAstructure\_tar\_file\_name*.    - A folder called RNAstructure will now be in the same directory as the tarball. The tarball can be deleted if desired.    - The GUI files are in the RNAstructure/exe directory, and can be run directly from there.    - Also, please refer to the Running the RNAstructure JAVA GUI page for instructions on how to start the GUI.    - The online help for the GUI is available here. It can also be accessed from the help menu item from within the program.   To install the text interfaces, follow these steps:   1. Download the text interfaces for either 32 or 64-bit from the Mathews Lab RNAstructure page. 2. The text interfaces are supplied as a GZipped tarball, so the files must be extracted. Move the tarball to the desired directory and execute the command *tar -xzvf RNAstructure\_tar\_file\_name*. 3. A folder called RNAstructure will now be in the same directory as the tarball. The tarball can be deleted if desired. 4. The text interface executables are in the RNAstructure/exe directory, and can be run directly from there. 5. Before running the text interfaces, please refer to command line help page for specifying the location of the thermodynamic parameters. 6. The online help for the command line programs is available here. | | |
| Visit The Mathews Lab RNAstructure Page for updates and latest information. | | |
